# Designing multi-epitope vaccine against important colorectal cancer (CRC) associated pathogens based on immunoinformatics approach

**DOI:** 10.1186/s12859-023-05197-0

**Published:** 2023-02-24

**Authors:** Hamid Motamedi, Marzie Mahdizade Ari, Mohsen Shahlaei, Sajad Moradi, Parisa Farhadikia, Amirhoushang Alvandi, Ramin Abiri

**Affiliations:** 1 Department of Microbiology, School of Medicine, Kermanshah University of Medical Sciences, Kermanshah, Iran; 2 Student Research Committee, School of Medicine, Kermanshah University of Medical Sciences, Kermanshah, Iran; 3 Department of Microbiology, School of Medicine, Iran University of Medical Sciences, Tehran, Iran; 4 Microbial Biotechnology Research Centre, Iran University of Medical Sciences, Tehran, Iran; 5 Nano Drug Delivery Research Center, Health Technology Institute, Kermanshah University of Medical Sciences, Kermanshah, Iran; 6 Medical Technology Research Center, Health Technology Institute,, Kermanshah University of Medical Sciences, Kermanshah, Iran; 7 Fertility and Infertility Research Center, Health Technology Institute,, Kermanshah University of Medical Sciences, Kermanshah, Iran

**Keywords:** Colorectal cancer, Gut microbiota, Immunoinformatics, Vaccine design, In silico, Multi-epitope vaccine

## Abstract

**Background:**

It seems that several members of intestinal gut microbiota like *Streptococcus bovis, Bacteroides fragilis, Helicobacter pylori, Fusobacterium nucleatum, Enterococcus faecalis, Escherichia coli, Peptostreptococcus anaerobius* may be considered as the causative agents of Colorectal Cancer (CRC). The present study used bioinformatics and immunoinformatics approaches to design a potential epitope-based multi-epitope vaccine to prevent CRC with optimal population coverage.

**Methods:**

In this study, ten amino acid sequences of CRC-related pathogens were retrieved from the NCBI database. Three ABCpred, BCPREDS and LBtope online servers were considered for B cells prediction and the IEDB server for T cells (CD4^+^ and CD8^+^) prediction. Then, validation, allergenicity, toxicity and physicochemical analysis of all sequences were performed using web servers. A total of three linkers, AAY, GPGPG, and KK were used to bind CTL, HTL and BCL epitopes, respectively. In addition, the final construct was subjected to disulfide engineering, molecular docking, immune simulation and codon adaptation to design an effective vaccine production strategy.

**Results:**

A total of 19 sequences of different lengths for linear B-cell epitopes, 19 and 18 sequences were considered as epitopes of CD4^+^ T and CD8^+^ cells, respectively. The predicted epitopes were joined by appropriate linkers because they play an important role in producing an extended conformation and protein folding. The final multi-epitope construct and Toll-like receptor 4 (TLR4) were evaluated by molecular docking, which revealed stable and strong binding interactions. Immunity simulation of the vaccine showed significantly high levels of immunoglobulins, helper T cells, cytotoxic T cells and INF-γ.

**Conclusion:**

Finally, the results showed that the designed multi-epitope vaccine could serve as an excellent prophylactic candidate against CRC-associated pathogens, but in vitro and animal studies are needed to justify our findings for its use as a possible preventive measure.

## Background

**Fig. 1:**
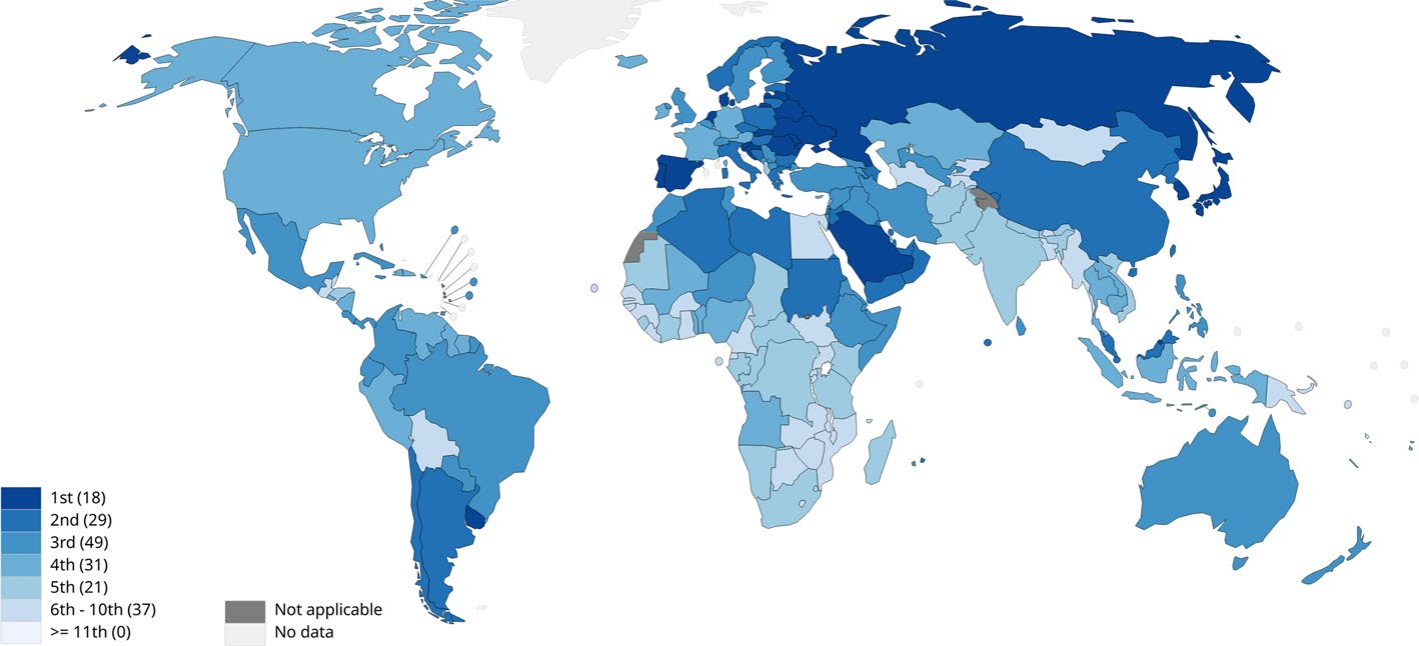
Incidence rank of CRC according to WHO reports (https://gco.iarc.fr/today)

The human gut microbiome contains 10^13^–10^14^ bacterial cells which play important roles in health and disease prevention. These functions consist of providing an energy supply, balancing immune responses, preventing pathogens' colonization and maintenance of intestinal epithelium integrity [1]. Microbiome dysbiosis or any change in the composition of the human microbiome is the result of environmental factors [2] like diet, antibiotic treatment and recurrent infections which may lead to physiological and pathological alterations [3, 4]. Colorectal cancer (CRC) refers to a genetic disorder with uncontrolled proliferation of colorectal epithelial cells and may be triggered or exacerbated by microbiome dysbiosis. CRC has the first rank in terms of incidence and second in terms of mortality in both females and males among all cancers [5, 6]. Figure 1 shows the latest update (2020) of the CRC incidence rates according to the World Health Organization (WHO) report. CRC has a complicated etiology, while several cases of cancer have inherited and genetic backgrounds, most CRC cases arise due to predisposing environmental factors [1, 7]. According to the reports of the national cancer institute, other risk factors for CRC are personal history of colorectal adenomas, previous colorectal or ovarian cancer, familial adenomatous polyposis (FAP) and Lynch syndrome (hereditary nonpolyposis colorectal cancer [HNPCC]), personal history of long-term chronic ulcerative colitis or Crohn colitis, heavy alcohol consumption, smoking, special race/ethnicities and obesity [5, 8–13].

A remarkable result of intestinal dysbiosis is the replacement of commensal microorganisms with potential pathogens. Seven potential pathogens including *Streptococcus bovis* (*S. bovis*) [14, 15], *Helicobacter pylori (H. pylori), Bacteroides Fragilis* (*B. Fragilis*), *Fusobacterium nucleatum* (*F. nucleatum*) [16], *Enterococcus faecalis* (*E. faecalis*), *Escherichia coli* (*E. coli*) [17, 18], and *Peptostreptococcus anaerobius* (*P. anaerobius*) [19, 20] are eminent microorganisms involved in the occurrence of CRC [14].

The relationship between bacteria and malignancies is complex. In many cases, oral or intestine resident bacteria prevent the development of cancer by stimulating immune response and production of anti-inflammatory compounds [21] such as IL-10 and bacterial metabolites such as single chain fatty acids (SCFAs like butyrate and propionate) [22, 23], Lipopolysaccharide (LPS) in gram-negative bacteria [24] and ferrichrome in *Lactobacillus casei* [25]. On other hand, the importance of bacteria in inducing cancers such as *H. pylori* and gastric cancers is proven. *H. pylori* is carcinogenic by producing CagA and VacA toxins [26]. Inflammation processes triggered by the intestinal microorganisms can also cause cancer. These associations were observed in *F. nucleatum* and *P. anaerobius*, which colonize the oral cavity and can induce colorectal cancer by stimulating inflammation. Enterotoxigenic B. *Fragilis* (ETBF) stabilize intestinal colonization by biofilm formation and induce chronic inflammation and progression to cancers. *E. faecalis* and *E. coli* are transient members of the normal flora of the intestine, vagina and oral cavity. These species may cause CRC progression by producing virulence factors such as toxins and enzymes which cause chromosome instability in human chromosomes and cell cycle arresting in colon epithelial cells [27].

Histone-like protein A (HlpA) in *S. gallolyticus*, FadA, Fap2 and RadD in *F. nucleatum* [28], and PCWBR2 in *P. anaerobius* [1] are the main adhesins in CRC-related bacteria. Bacterial cell wall HlpA is the main surface immunogenic protein that enables *S. gallolyticus* to bind to Heparin sulfate proteoglycans (HSPGs) and stimulates a humoral immune response. Fap2 interacts and inactivates T lymphocytes in favor of tumor cell growth [29, 30]. RadD mediates biofilm formation and attachment of *F. nucleatum* cells to the host cells and the same as Fap2 supports the growth of tumor cells. It is also claimed that the interaction between the putative cell wall binding repeat 2 (PCWBR2) surface protein of *P. anaerobius* and α2/β1 integrin activates a signaling pathway associated with an increased risk of CRC [20]. Considering the potent immunogenic activity of the aforementioned proteins, they are potential vaccine candidates for the related bacteria causing CRCs.

Some bacterial protein toxins such as CagA and VacA produced by *H. pylori* [31–34], ETBF by *B. fragilis* and Cytolethal distending toxin (CDT) and Colibactin produced by *E. coli* are potent carcinogen promoters. They can elicit inflammatory reactions, interfere with signaling pathways and also may hamper cell cycles in favor of carcinogenesis. CagA and VacA toxins promote CRC during the reverse-feedback mechanism and hypergastrinemia and are known as the major factors of gastric cancer and possible inducers of CRC by affecting apoptosis and signal transduction systems of the cells, vacuolization and changing epithelial permeability, respectively [26, 35, 36]. ETBF is a zinc-dependent metalloprotease that cleaves E-cadherin molecules, and its interactions with intestinal epithelial cells lead to the activation of the STAT3 pathway. The toxin causes IL-2 reduction and IL-17 increase, which lead to the proliferation and survival of cancer cells [37, 38]. CDT, cytotoxic necrotizing factor (CNF), cycle inhibiting factor, Shiga toxin and subtilase toxin are important cyclomodulins which can alter the cell cycle in favor of bacterial invasion and colonization [39]. CDT and Colibactin arrest the cell cycle and damage the double-stranded structure of DNA by alkylating adenine bases [40]. Superoxide dismutase (SOD) as a virulence factor of *E. faecalis* induce damage in the DNA backbone and predisposes the colon epithelial cells to mutations and cancer [41–43]. To the best of our knowledge, no study is conducted on multi-epitope vaccines against different CRC-inducing pathogens, so the present study is the first report intending to design a multi-epitope vaccine based on in silico designing and immunoinformatics approach against the most important CRC-related bacterial pathogens.

## Methods

**Fig. 2:**
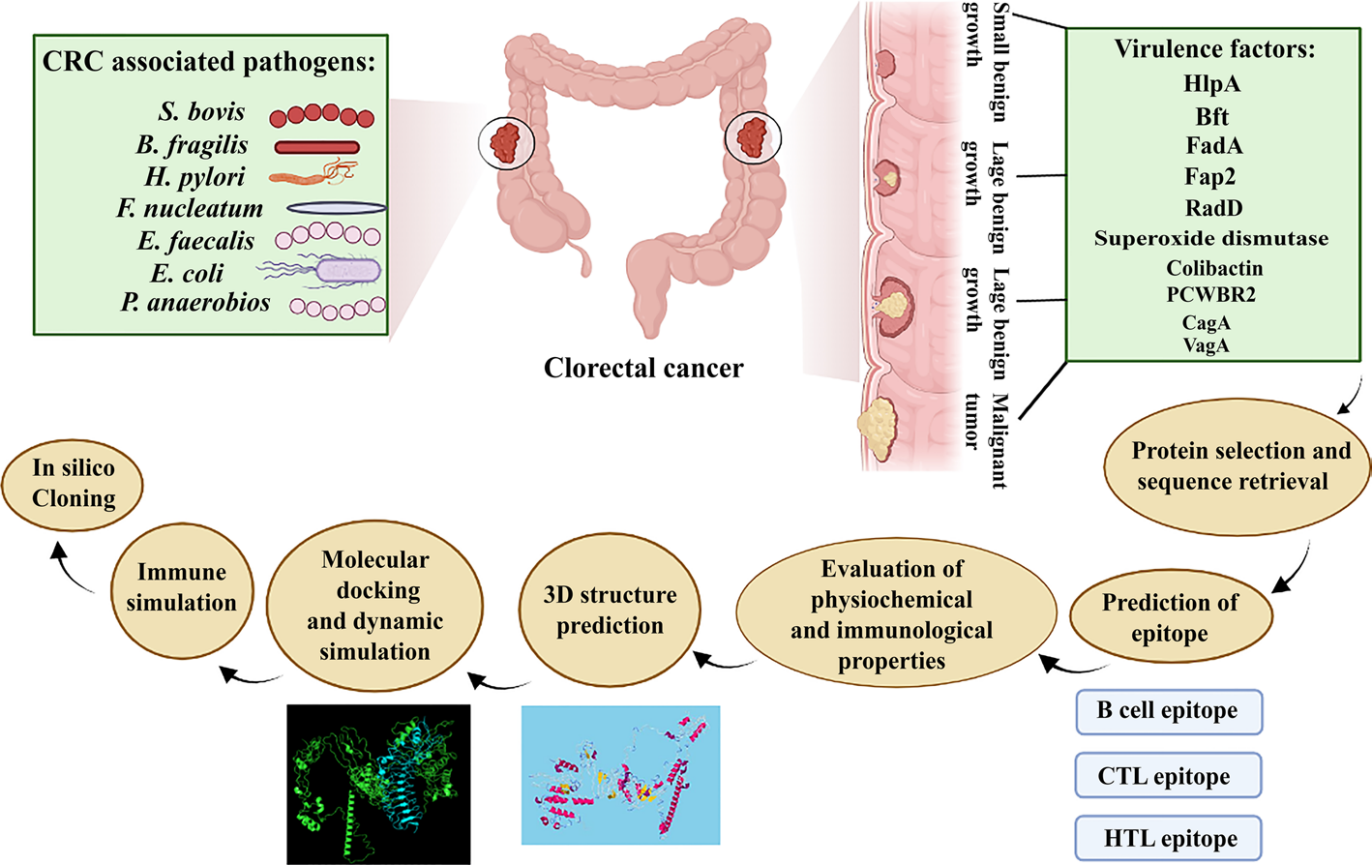
An overview of the steps of making a multi-epitope vaccine by in silico method in the present study

In order to design multi-epitope vaccine against CRC-promoting bacteria, CD4^+^ and CD8^+^ T cell and B cell stimulating epitopes were selected. Then, validation, allergenicity, toxicity and physicochemical properties of all epitopes were performed using different web servers. Three linkers AAY, GPGPG and KK were used to connect cytotoxic T cell epitope (CTL), T-helper lymphocyte (HTL) and B-cell lymphocyte (BCL) epitopes, respectively. For assessment of the stability and binding affinity, the TLR4 receptor was docked by ligands using FireDock, PatchDock and ClusPro 2.0 servers. Finally, codon adaptation and in silico cloning studies were carried out. In addition, the C-ImmSim server was used to describe the humoral and cellular profile of the mammalian immune system against the designed vaccine. The workflow for this scientific study is shown in Fig. 2.

### Retrieval of bacterial sequences associated with CRC

In this study, ten proteins including HlpA, BFT, (FadA, Fap2 and RadD), Superoxide, Colibactin, PCWBR2 and (CagA and VacA) were selected for design of multi-epitope vaccines against *S. gallolyticus,* ETBF*, F. nucleatum, E. faecalis, E. coli, P. anaerobius* and* H. pylori,* respectively. The related sequences were retrieved from the National Center for Biotechnology Information (NCBI) Protein Database (https://www.ncbi.nlm.nih.gov/protein/?term=) database (Table 1).

**Table 1:** Details of sequences retrieved from CRC cancer related pathogens

Organism	Protein	Accession numbers	Amino acids
*Streptococcus gallolyticus*	HlpA	KJF00052.1	91 aa
*Bacteroides fragilis*	Bft	AAB50410.2	389 aa
*Fusobacterium nucleatum*	FadA	AAY47043.1	129 aa
	Fap2	WP_059222898.1	3784 aa
	RadD	WP_238968484.1	3463 aa
*Enterococcus faecalis*	Superoxide dismutase	EPI15045.1	202 aa
*Escherichia coli*	Colibactin	WP_193793145.1	3206 aa
*Peptostreptococcus anaerobius*	PCWBR2	KXI10301.1	376 aa
*Helicobacter pylori*	CagA	P55980.1	1186 aa
	VacA	AAU85846.1	746

### Prediction of B cell epitopes

B cell epitopes play a pivotal role in the development of peptide vaccines, in the diagnosis of diseases as well as for allergy research [44]. Three servers, ABCpred (https://webs.iiitd.edu.in/raghava/abcpred/ABC_submission.html), BCPREDS (http://ailab-projects1.ist.psu.edu:8080/bcpred/) and LBtope (https://webs.iiitd.edu.in/raghava/lbtope/protein.php), were used to predict B cell epitopes. We applied three different servers to obtain the best coverage of the predicted epitopes. ABCpred is developed based on a recursive neural network (machine-based technique) using a fixed length pattern and can predict epitopes with 65.93% accuracy using this network [44]. The BCPREDS server uses three developed methods, AAP, BCPred and FBCPred, to predict B cell epitopes [45, 46]. The server, on the other hand, uses a support vector machine (SVM) algorithm to predict B-cell (linear) epitopes. ABCpred and BCPREDS servers have variable epitope lengths (10–20) and (12–22) to predict B cell epitopes, respectively. The third server used to predict B cell was the LBtope server [47]. Due to the high accuracy of epitope prediction, we considered the cut-offs above 0.6 to predict B cell epitopes in this server.

### MHC-I binding epitopes (CTL) prediction

Determination of peptide binding to major histocompatibility complex (MHC) class I is an important step in CTL detection methods for MHC class I peptide binding [48]. All 10 proteins in this study were screened for MHC-I (18 HLA-A, 32 HLA-B and 20 HLA-C) alleles based on the Immune Epitope Database server (IEDB; http://tools.iedb.org/mhci/) [49]. Length preferences can vary depending on the MHC allele but are generally limited to peptides of length 8–11 amino acids [50]. We considered 9-mer epitopes, NetMHCpan EL 4.1 method and a score above 0.5 to predict the desired epitopes.

### MHC class II binding prediction

HLA class II molecules are expressed by human antigen-presenting cells (APCs) and are used directly to identify epitope candidates in infectious agents, allergens, cancer, and autoantigens [51]. The IEDB (http://tools.iedb.org/mhcii/) was used to predict HTL epitopes for 10 proteins. The IEDB parameters used for this study included a selection of peptide length 15 mer, IEDB recommended 2.22, and human Leukocyte Antigen (HLA) reference set (containing 27 alleles). The selection IEDB Recommended uses the Consensus approach, combining NN-align, SMM-align, CombLib and Sturniolo if any corresponding predictor is available for the molecule, otherwise, NetMHCIIpan is used [52]. Finally, to predict epitopes MHC class II with high binding power, we used adjusted rank < 2 to filter.

### Vaccine construction

In the present study, CTL, HTL, and BCL were joined together with a suitable linker to make an effective multi-epitope vaccine. A total of three linkers, AAY, GPGPG, and KK were used to bind CTL, HTL and BCL epitopes, respectively. The reason for using these linkers is that they play a vital role in producing a wide conformation (flexibility), protein folding and separation of functional domains, and therefore they are able to make the protein structure more stable.

### Prediction of various physicochemical properties

The study of physical and chemical properties reveals the functional and structural properties of a protein. ProtParam (http://web.expasy.org/protparam/) server was used to evaluate the physical and chemical properties of the final vaccine construct [53]. This server has various physical and chemical parameters of proteins such as amino acid composition, extinction coefficient, instability index, total hydropathic mean (GRAVY), aliphatic index, theoretical pI, atomic composition and molecular weight allowing us to understand the stability, activity and nature of proteins [54]. The instability index (II) of a protein indicates its stability of the protein. If the calculated protein instability index is less than 40, it was considered as a stable protein. GRAVY is used to indicate the hydrophobicity value of a peptide that calculates the sum of the hydropathic values of all amino acids divided by the length of the sequence [55]. In addition, the Aliphatic index (AI) is defined as the relative volume of protein occupied by its aliphatic side chains that are involved in the thermal stability of a protein.

Another important feature that should be considered in vaccine design is protein solubility, which is important in industrial and therapeutic applications. In this study, the Protein-Sol web server was used to predict protein solubility [56]. If our protein solubility score (scaled solubility value or QuerySol) was less than 0.45, it indicates that our protein is more soluble than the average soluble *E. coli* protein.

### Identifying antigenicity, allergenicity and toxicity of protein sequences

The VaxiJen server (http://www.ddg-pharmfac.net/vaxijen/VaxiJen/VaxiJen_citation.html) is the first server for alignment-independent prediction of protective antigens [57]. This server examines bacterial, viral, and tumor protein datasets to predict protein antigenicity. In addition, it has shown a prediction accuracy of 70 to 89 percent [57]. In this study, our target organism was bacteria and other parameters were selected by default.

Allergy is a harmful consequence of a wrong immune response that has evolved to develop immunity to macroparasites [58]. The AllerTOPv.2 server (https://www.ddg-pharmfac.net/AllerTOP/) is used to predict allergenicity [59]. On the other hand, AllerTOP is known as the first suitable alignment-free server for in silico prediction of allergens based on the physicochemical properties of protein sequences. Version 2 of this server is a significant improvement over version 1 and has an accuracy of 88.7%. It is also highly sensitive (94%) compared to other allergenic prediction servers [59, 60]. ToxinPred (http://crdd.osdd.net/raghava/toxinpred/multi_submit.php) is a unique method in silicon that will be useful in predicting the toxicity of peptides/proteins, designing toxic peptides and detecting toxic regions in proteins [61].

### Population coverage of epitopes

T cells detect a complex between a specific molecule of MHC and a specific pathogen-derived epitope [62]. Specific HLA alleles are expressed with very different frequencies in different ethnicities. Therefore, the IEDB population coverage server (http://tools.iedb.org/population/) was used in the design and development of T-cell epitope-based vaccines for population coverage analysis [62]. In this study, population coverage for a vaccine designed in both MHC Class I and MHC Class II types in different ethnicities was examined.

### Secondary and tertiary structure prediction of the vaccine construct

One of the most important and challenging issues in the field of bioinformatics is the prediction of the secondary structure of the protein [63]. The secondary structure refers to the polypeptide backbone of local conformation proteins, which consists of three parts: regular secondary structure, α-helix and β-strand, and a type of irregular secondary structure, the coil region [63, 64]. In this study, PSIPRED (http://bioinf.cs.ucl.ac.uk/psipred/) tool was used to predict the secondary structure. It is one of the most widely used servers that use two feed-forward neural networks to analyze the output obtained from PSI-BLAST [65].

The RaptorX server (http://raptorx.uchicago.edu/) was used to model the 3-dimensional (3D) structure. RaptorX offers high-quality structural models (5 models) for many purposes. It also takes the server about 35 min to complete the processing of a sequence of 200 amino acids. The server, on the other hand, is designed for protein secondary structure prediction, alignment quality assessment and sophisticated probabilistic alignment sampling [66].

### Refinement, validation and quality assessment of the 3D structure

The importance of improving template-based model structures beyond the existing accuracy of template information in the structure prediction community is emphasized. For this reason, the GalaxyRefine server (http://galaxy.seoklab.org/cgi-bin/submit.cgi?type=REFINE) was used to refine the structure of the protein model [67]. GalaxyRefine server has different parameters that include: global distance test-high accuracy (GDT-HA), root-mean-square deviation (RMSD), MolProbity, and Ramachandran favored score. MolProbity shows crystallographic resolution and typical scores for experimental structures range from 1 to 2. RMSD is the most commonly used quantitative measure of the similarity between two superimposed atomic coordinates [68]. A lower RMSD value indicates better stability, and an RMSD score between 0 and 1.2 Angstrom (Å) is usually acceptable.

UCLA-DOE LAB (https://saves.mbi.ucla.edu/) and ProSA-web (https://prosa.services.came.sbg.ac.at/prosa.php) servers were used to evaluate the validity and quality of the selected 3D structure [69, 70]. The UCLA-DOE LAB server has various tools such as PROCHECK and ERRAT for 3D structure validation. Ramachandran diagram was analyzed using the PROCHECK section from the UCLA-DOE LAB server (http://molprobity.manchester.ac.uk/). The Ramachandran diagram shows the statistical distribution of the combination of the backbone dihedral angles φ and ψ, as well as the percentage and number of residues in the most favored, additional allowed, generously allowed, and disallowed region, which defines the quality of modeled structure [71].

ProSA-web server (https://prosa.services.came.sbg.ac.at/prosa.php) is a tool used to study 3D models of protein structures for possible errors [70]. One of the structural features derived from this server is the z-score. The z-score indicates overall model quality and measures the deviation of the total energy of the structure concerning an energy distribution derived from random conformations [72]. In addition, a plot of local quality scores points to problematic parts of the model which are also highlighted in a 3D molecule viewer to facilitate their detection [70].

### Multi-epitope vaccine protein disulfide engineering

Disulfide bridges are formed between cysteine residues in peptides and proteins and are recognized as an essential element in the molecular architecture of proteins and peptides [73]. It is also believed that these bonds reduce conformational entropy and increase the free energy of the denatured state, thus increasing the stability of the protein structure [74]. In the present study, Disulfide by Design 2.0 (DbD2) (http://cptweb.cpt.wayne.edu/DbD2/) online server was used to detect disulfide bonds [75]. The server can provide refined 3D structures of the vaccine to identify residual pairs that can form disulfide bonds. When engineering the disulfide bonds, the intra-chain, inter-chain and C_β_ for glycine residue, were selected and the v3 and Cα-C_β_-Sγ angles were kept at − 87° or + 97° ± 30 and 114.6° ± 10, respectively. Finally, an energy value of less than 1 kcal/mol was selected as the threshold for the remaining pair [75]. Because 90% of native disulfide bonds usually have an energy value of less than 2.2 kcal/mol [76].

### Molecular docking of multi-epitope vaccine with TLR4

Docking is recognized as an important tool in computer-aided drug design. Protein–protein docking analysis was performed through the ClusPro 2.0 server [77]. This server requires two receptor and ligand files in the form of PDB. TLR-4 acts as a receptor for antigen recognition, which plays a role in immune activation and mediating cytokine induction [78]. The results obtained from this server include rigid body connection, clustering of the lowest energy structure, and structural refinement by minimizing energy. The vaccine-ligand complex was obtained based on the lowest energy and docking efficiency.

Docking analysis was again used by the PatchDock server (https://bioinfo3d.cs.tau.ac.il/PatchDock/) [79] to confirm the affinity of the vaccine structure designed with TLR4. The PatchDock server predicted potential complexity using three algorithm-molecular shape representations, surface patch matching, filtering, and scoring. Consequently, the top 10 results of the PatchDock server were evaluated using the FireDock (https://bioinfo3d.cs.tau.ac.il/FireDock/) server [80] to calculate the Global binding energy that consists of attractive and repulsive van der Waals (VdW) forces, atomic contact energy (ACE) and hydrogen bond. Before the docking process, the H2O molecules, ligands and polar hydrogens were removed while the Kollman charge was added. The structural coordinates of TLR4 were retrieved from the Protein Data Bank (PDB) (https://www.rcsb.org/) using the respective PDB ID: 2Z62. Finally, the visualization of complex vaccine-TLR interactions was performed by LigPlot + software.

### Molecular dynamics simulation

The dynamic stability of the designed vaccine was investigated by performing a 100 ns molecular dynamics (MD) simulation. MD was performed using GROMACS package v2020, which provides a rich set of computational and analysis tools [81]. The parameters for MD simulation were derived from Amber sb99 force field and the system was the solvated by SPC/E water model. After electro-neutralization of the solvated simulation box the energy minimization was performed by the aim of steepest descend algorithm. Temperature and pressure were adjusted at 310 k and 1 bar respectively using a nose–hoover thermostat and a Parinello- Rahman barostat. All bonds were constrained by LINear Constraint Solver (LINCS) method. Both van der Waals and electrostatic non-bonded interactions were measured by the cutoff of 1 nm. In this regards the long range electrostatics were treated by PME method. Finally, a 100 ns MD simulation was carried out under the leap-frog algorithm.

### Normal mode analysis (NMA)

The study of molecular dynamics (MD) is essential to evaluate the stability and physical motility of the vaccine-TLR4 docked complex in any in silico assay. Therefore, protein stability can be determined by comparing the dynamics of essential proteins with their normal modes [81, 82]. To perform the molecular dynamics simulation process, an iMODS server (http://imods.chaconlab.org/) based on a normal state analysis (NMA) conductor was used [82–84]. Then, the complex of vaccine construct-TLR was delivered to the iMODS server. This iMODS server evaluates the stability of a protein by calculating its NMA. The server also provides images of factor B-factor and deformability plots, covariance map, mode variance plot, eigenvalues and elastic networks.

### Immune simulation

C-IMMSIM server (http://kraken.iac.rm.cnr.it/C-IMMSIM/index.php?page=1) is an agent-based simulator of the immune response that uses bioinformatics methods to predict T and B cell epitopes [85]. The C-ImmSim utilizes the Celada-Seiden model for describing both humoral and cellular profiles of a mammalian immune system against a designed vaccine. In summary, this server /C-IMMSIM displays images in which the major classes of cells of both the lymphoid [T helper lymphocytes (Th)], CTL, B lymphocytes, and antibody-producer plasma cells, PLB) and the myeloid lineage [macrophages (M) and dendritic cells] are represented [85]. The simulated parameters in this study included: (a) a vaccine without LPS, (b) considering three doses of vaccine (to create an efficient and long-lasting immune response) with time intervals of 1, 84 and 168 days, (c) the volume of the simulation and the simulation steps were adjusted to 10 and 1100, respectively. The other parameter "Random Seed" remains unchanged. It should be noted that one step of the simulation is equivalent to eight hours (8 h) of real-time, allowing immune response modeling for about 350 days [i.e. (1050 × 8 h)/(24 h)].

### Codon-optimization and cloning for the design of multi-epitope vaccine

Today, we need a set of predictor servers to adapt the usage of the target gene codon for most sequenced prokaryotes and the eukaryotic gene expression hosts selected to improve heterologous protein production [86]. Java Codon Adaptation Tool (JCat) server (http://www.jcat.de/) was used to quantify the expression level of the multi-epitope vaccine in *E. coli* (strain K12). This server calculates two important outputs for the query sequence to ensure maximum expression. One of them is GC content and the other is Codon Adaptation Index (CAI) value [86]. CAI requires the definition of high-expression genes that allow a comparable value to be calculated for codon usage. Finally, the vaccine construct was cloned into plasmid pET-28a (+) using SnapGene software (version 5.2.3) (https://www.snapgene.com/).

### Analysis of the vaccine MRNA

The Vienna RNA website is known as a comprehensive collection of tools for folding, designing and analyzing RNA sequences [87]. In this study, RNAfold (http://rna.tbi.univie.ac.at/cgi-bin/RNAWebSuite/RNAfold.cgi) web server was used to predict the secondary structure of MRNA. At this stage, after obtaining the optimized DNA sequence through the JCat server, for analysis of MRNA folding and vaccine secondary structure, first converted into a potential DNA sequence by DNA <—> RNA- > Protein at (http://biomodel.uah.es/en/lab/cybertory/analysis/trans.htm). Finally, the minimum free energy (MFE) score was important to us. MFE of ribonucleic acids (RNAs) increases at an apparent linear rate with sequence length. Simple indices, obtained by dividing the MFE by the number of nucleotides, have been used for a direct comparison of the folding stability of RNAs of various sizes [88].

## Results

### Retrieval of bacterial sequences associated with colorectal cancer

Ten protein sequences with different amino acid lengths from colorectal cancer-related pathogens were retrieved in the FASTA format.

### Prediction of B cell epitopes

The reason for examining B-cell epitopes is their extraordinary ability to neutralize pathogenic molecules through the secretion of antibodies [89, 90]. ABCpred, BCPREDS and LBtope servers were used for B-cell prediction. Preliminary analysis showed that a total of 19 epitopes with criteria such as antigenic, non-allergenic and non-toxic were selected (Table 2). It should be noted that epitopes were considered for the final vaccine that overlapped at least two or three high-score servers.

**Table 2:** Prediction of B cell epitopes based on ABCpred, LBtop and BCPREDS servers

Protein	Length	Peptide	Start	ABCpred	LBtop	BCPREDS	Antigenicity	Allergenicity	Toxicity
HlpA	12	NFEVRERAARKG	50	0.54	–	0.99	1.7361	NON-ALLERGEN	Non-Toxin
BFT	14	SLKANPKAEGYDDQ	278	0.74	–	0.905	1.2	NON-ALLERGEN	Non-Toxin
	20	TEYSCPSGNADEGLDGFTAS	259	0.87	0.64	–	0.9369	NON-ALLERGEN	Non-Toxin
FadA	12	SQYQDLASKYED	89	0.7	0.6	–	0.5765	NON-ALLERGEN	Non-Toxin
	18	LDAEYQNLANQEEARFNE	32	0.78	0.61	–	0.7945	NON-ALLERGEN	Non-Toxin
Fap2	12	DGASTNPDPNKL	2518	–	0.77	0.999	1.033	NON-ALLERGEN	Non-Toxin
	18	EEVNLENSQVATREELKT	42	0.87	0.66	0.928	1.1408	NON-ALLERGEN	Non-Toxin
RadD	12	EGTNNEVDHNTD	1612	0.72	68.65	0.986	1.621	NON-ALLERGEN	Non-Toxin
	14	DLGTIDFNGDDGVG	1222	0.76	70.12	0.974	1.3681	NON-ALLERGEN	Non-Toxin
Superoxide	16	HPELGEKSVEDLISDM	46	0.81	0.62	–	0.5832	NON-ALLERGEN	Non-Toxin
	20	IPEDIRTAVRNNGGGHANHT	64	–	0.61	0.882	1.0386	NON-ALLERGEN	Non-Toxin
Colibactin	16	LEAHQHEDDPSATGVR	1503	–	0.64	1	1.3	NON-ALLERGEN	Non-Toxin
	20	QPPEGESNAPSPQPAVQTNT	3163	–	0.78	1	1	NON-ALLERGEN	Non-Toxin
PCWBR2	12	INKLNVSRISGK	70	0.64	0.7	–	0.7269	NON-ALLERGEN	Non-Toxin
	18	RYETSVKVSDELQKMSSG	83	0.78	0.65	–	0.9622	NON-ALLERGEN	Non-Toxin
CagA	12	NASKNPNKGVGA	515	–	0.69	0.99	1.1717	NON-ALLERGEN	Non-Toxin
	14	ESSTKSFQKFGDQR	108	0.74	0.65	0.89	0.6742	NON-ALLERGEN	Non-Toxin
VacA	14	VGGYKASLTTNAAH	408	–	67.3	0.95	0.8495	NON-ALLERGEN	Non-Toxin
	20	NFEFKAGTDTKNGTATFNND	472	0.87	0.67	0.95	1.5291	NON-ALLERGEN	Non-Toxin

### MHC-I binding epitopes (CTL) prediction

The MHC-I binding epitopes (9 mer) predicted by the IEDB recommended method for 70 available alleles (including 18 HLA-A, 32 HLA-B, and 20 HLA-C) were performed by the IEDB server. Among a large number of MHC-I predicted epitopes, 18 epitopes were selected as vaccine candidates. The selection of epitopes based on characteristics such as high score (good binder), antigenic, non-allergenic and non-toxic is shown in Table 3.

**Table 3:** Most probable predicted epitopes with MHC class I alleles from IEDB analysis tool

Protein	Peptide sequence	Start	End	Allele	Score	Antigenicity	Allergenicity	Toxicity
HlpA	SAAAVDAVF	22	30	HLA-B*35:01	0.861527	0.5365	NON-ALLERGEN	Non-Toxin
Bft	KANPKAEGY	280	288	HLA-A*30:02	0.830051	1.0961	NON-ALLERGEN	Non-Toxin
				HLA-B*15:01	0.665572			
				HLA-B*57:01	0.615497			
				HLA-B*58:01	0.596159			
	YPGVMAHEL	333	342	HLA-B*35:01	0.819059	0.6723	NON-ALLERGEN	Non-Toxin
				HLA-B*07:02	0.718513			
				HLA-B*53:01	0.716264			
				HLA-B*51:01	0.59173			
				HLA-B*08:01	0.523581			
FadA	QVYNELSQR	66	74	HLA-A*68:01	0.957771	0.8312	NON-ALLERGEN	Non-Toxin
				HLA-A*31:01	0.795891			
				HLA-A*11:01	0.657712			
				HLA-A*33:01	0.58309			
				HLA-A*03:01	0.508062			
Fap2	KTISVTAEK	1975	1983	HLA-A*11:01	0.978654	0.7777	NON-ALLERGEN	Non-Toxin
				HLA-A*03:01	0.956606			
				HLA-A*30:01	0.866036			
				HLA-A*68:01	0.820562			
				HLA-A*31:01	0.691021			
	FADGLEQRY	3426	3434	HLA-A*01:01	0.972812	1.1206	NON-ALLERGEN	Non-Toxin
				HLA-B*35:01	0.923357			
				HLA-B*53:01	0.519186			
RadD	GANPSVEYW	304	312	HLA-B*58:01	0.991946	1.1284	NON-ALLERGEN	Non-Toxin
				HLA-B*57:01	0.987697			
				HLA-B*53:01	0.73539			
	KEQENISQM	58	66	HLA-B*40:01	0.954627	0.4345	NON-ALLERGEN	Non-Toxin
				HLA-B*44:03	0.828703			
				HLA-B*44:02	0.783902			
superoxide dismutase	YIDVETMHL	18	26	HLA-A*02:06	0.78953	1.3306	NON-ALLERGEN	Non-Toxin
				HLA-A*02:01	0.737224			
	TPVLGLDVW	158	166	HLA-B*53:01	0.930286	1.9457	NON-ALLERGEN	Non-Toxin
				HLA-B*35:01	0.50924			
Colibactin	YLDALAQQL	2339	247	HLA-A*02:01	0.981781	0.4388	NON-ALLERGEN	Non-Toxin
				HLA-A*02:06	0.926393			
				HLA-A*02:03	0.777941			
	KADLAQLRY	970	978	HLA-A*01:01	0.970875	0.9347	NON-ALLERGEN	Non-Toxin
				HLA-A*30:02	0.650643			
				HLA-B*58:01	0.611357			
PCWBR2	YIIQNIYLV	149	157	HLA-A*02:06	0.914671	0.6827	NON-ALLERGEN	Non-Toxin
				HLA-A*02:01	0.884041			
				HLA-A*02:03	0.70463			
				HLA-A*68:02	0.587899			
	RVDTAFAVY	187	195	HLA-A*01:01	0.860066	0.4398	NON-ALLERGEN	Non-Toxin
				HLA-A*30:02	0.801369			
				HLA-B*15:01	0.533769			
CagA	VPASLSAKL	1050	1058	HLA-B*07:02	0.925163	1.1446	NON-ALLERGEN	Non-Toxin
				HLA-B*35:01	0.668443			
				HLA-B*53:01	0.618146			
				HLA-B*51:01	0.60881			
	GINPEWISK	735	743	HLA-A*11:01	0.904802	1.2858	NON-ALLERGEN	Non-Toxin
				HLA-A*03:01	0.83302			
VacA	GEKLVIDEF	600	608	HLA-B*44:03	0.863097	0.4654	NON-ALLERGEN	Non-Toxin
				HLA-B*44:02	0.801589			
				HLA-B*40:01	0.72177			
	RVNNQVGGY	456	464	HLA-A*30:02	0.84623	1.5337	NON-ALLERGEN	Non-Toxin
				HLA-B*15:01	0.597925			

### MHC class II binding prediction

MHC-II binding epitopes (15 mer) were examined for 27 alleles (including HLA-DR, HLA-DQ, and HLA-DP) using the IEDB-recommended method. From a large number of HTL epitopes, we selected 19 epitopes with a length of 15 amino acids, which are shown in Table 4. The criteria for selecting these epitopes were low adjusted rank (good binder), antigenic, non-allergenic and non-toxic properties.

**Table 4:** Most probable predicted epitopes with MHC class II alleles from IEDB analysis tool

Protein	Peptide sequence	Start	End	Allele	Score	Antigenicity	Allergenicity	Toxicity
HlpA	KKDSAAAVDAVFSAI	19	33	HLA-DQA1*04:01/DQB1*04:02	2.5	0.5391	NON-ALLERGEN	Non-Toxin
Bft	SFILGDEFAVLRFYR	94	108	HLA-DPA1*01:03/DPB1*04:0	0.24	0.4059	NON-ALLERGEN	Non-Toxin
				HLA-DPA1*02:01/DPB1*01:01	0.29			
				HLA-DPA1*01:03/DPB1*02:01	0.97			
				HLA-DPA1*03:01/DPB1*04:02	1.3			
	HGLKRFVNLHFVLYT	244	258	HLA-DRB1*15:01	0.33	0.4609	NON-ALLERGEN	Non-Toxin
				HLA-DPA1*01:03/DPB1*04:01	1.4			
FadA	AVLAVSASAFAATDA	8	22	HLA-DQA1*03:01/DQB1*03:02	0.97	0.4315	NON-ALLERGEN	Non-Toxin
				HLA-DRB1*09:01	1.3			
				HLA-DQA1*05:01/DQB1*03:01	1.5			
	SLVGELQALDAEYQN	24	38	HLA-DQA1*05:01/DQB1*02:01	1.3	0.4966	NON-ALLERGEN	Non-Toxin
Fap2	AVLVANNGANVEIAS	1112	1126	HLA-DRB1*13:02	0.01	0.7538	NON-ALLERGEN	Non-Toxin
				HLA-DRB3*02:02	0.15			
				HLA-DQA1*01:02/DQB1*06:02	0.34			
	EKIKNLRLELIQLME	77	91	HLA-DRB4*01:01	0.21	0.6094		
				HLA-DPA1*03:01/DPB1*04:02	0.99			
				HLA-DPA1*02:01/DPB1*01:01	1.5			
RadD	LVKFNINATKAIGIL	599	613	HLA-DRB3*02:02	0.02	0.4117	NON-ALLERGEN	Non-Toxin
				HLA-DRB1*07:01	0.21			
				HLA-DRB1*13:02	0.89			
				HLA-DRB1*09:01	1.4		NON-ALLERGEN	Non-Toxin
				HLA-DPA1*02:01/DPB1*14:01	2			
	AKLINNMNVTVGVDA	2626	2640	HLA-DRB1*13:02	0.15	0.806		
				HLA-DRB3*02:02	0.3			
superoxide dismutase	ELPYAYDALEPYIDV	7	21	HLA-DRB3*01:0	0.93	0.4856	NON-ALLERGEN	Non-Toxin
				HLA-DQA1*05:01/DQB1*02:0	0.24			
				HLA-DQA1*01:01/DQB1*05:01	1.7		NON-ALLERGEN	Non-Toxin
	KAAFKTAATGRFGSG	116	130	HLA-DRB1*09:01	0.36	0.8373		
				HLA-DPA1*02:01/DPB1*14:01	0.53			
Colibactin	QKGFRFSIAYALNYL	425	438	HLA-DPA1*02:01/DPB1*14:01	0.01	1	NON-ALLERGEN	Non-Toxin
				HLA-DRB1*07:0	0.14			
				HLA-DRB3*02:02	0.33			
				HLA-DRB5*01:01	0.37		NON-ALLERGEN	Non-Toxin
				HLA-DPA1*01:03/DPB1*04:01	0.47			
				HLA-DRB1*01:01	1.6			
				HLA-DRB1*09:01	1.6			
	ALPIAYLTAYYALVV	2797	2811	HLA-DRB1*01:01	0.2	0.6023		
				HLA-DRB1*12:01	1.11			
				HLA-DRB1*01:01	1.8			
				HLA-DPA1*01:03/DPB1*04:01	0.56			
				HLA-DPA1*01:03/DPB1*02:01	1.2			
PCWBR2	DALTAGTMAAELEIP	115	129	HLA-DQA1*04:01/DQB1*04:02	0.61	0.8571	NON-ALLERGEN	Non-Toxin
				HLA-DQA1*01:02/DQB1*06:02	1.1			
				HLA-DQA1*03:01/DQB1*03:02	1.6			
	LEIPLLLTKSNKLPD	126	140	HLA-DRB1*15:01	0.84	0.4256		
CagA	FMEFLAQNNTKLDNL	404	418	HLA-DRB1*04:01	0.41	0.5405	NON-ALLERGEN	Non-Toxin
				HLA-DRB3*02:02	0.77			
				HLA-DRB3*02:02	0.54	0.5348		
				HLA-DRB1*13:02	0.7			
	YKFNQLLIHNNALSS	292	306	HLA-DRB1*04:01	1.3		NON-ALLERGEN	Non-Toxin
VacA	EYDLYKSLLSSKIDG	97	111	HLA-DRB1*01:01	0.19	0.6034	NON-ALLERGEN	Non-Toxin
				HLA-DRB1*07:01	0.69			
				HLA-DRB1*04:05	0.72			
	KLVIDEFYYSPWNYF	602	616	HLA-DRB1*04:01	0.94		NON-ALLERGEN	Non-Toxin
				HLA-DPA1*01:03/DPB1*04:01	0.61	0.6653		
				HLA-DPA1*01:03/DPB1*02:01	2			

### Prediction of various physicochemical properties

**Fig. 3:**
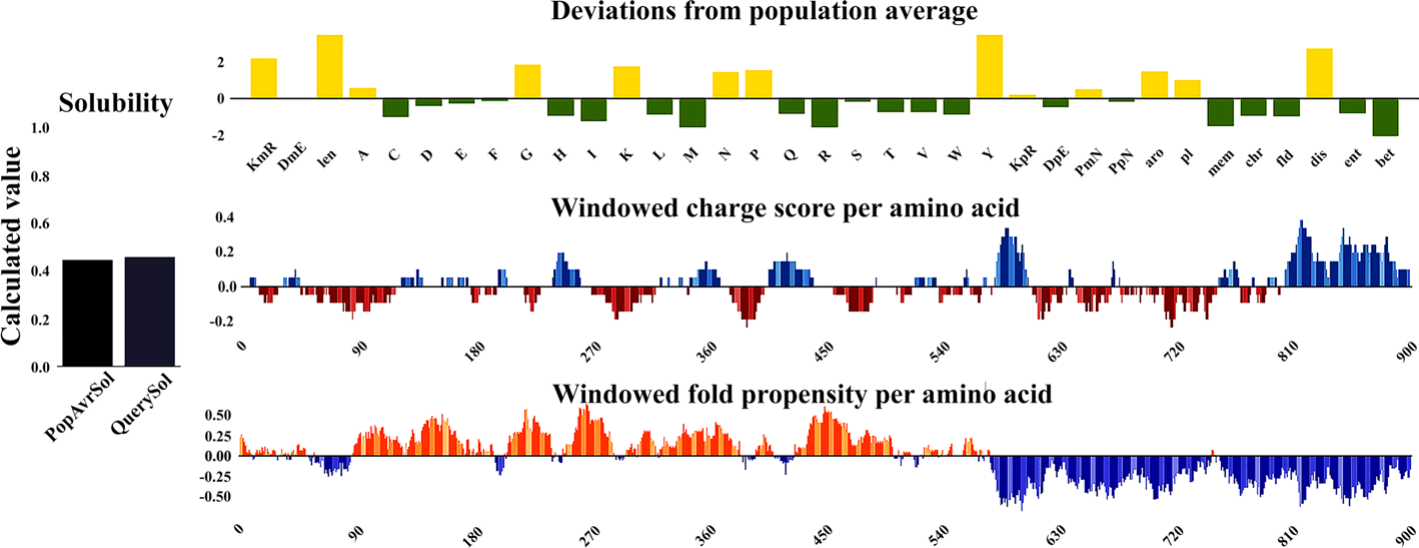
The solubility of the vaccine structure according to QuerySol was 0.460, which showed that it has good solubility

The final vaccine construct containing 924 amino acids and its molecular weight was determined based on ProtParam server 99 kDa. Since the final molecular weight of our final construct is less than 110 kDa, it can be considered a suitable vaccine [91]. The vaccine contained 105 (Arg + Lys) positively charged residues. The estimated half-life is 1.9 h (mammalian reticulocytes, in vitro), 20 h (yeast, in vivo), and more than 10 h (*E. coli*, in vivo). The vaccine construct was composed of 13,973 atoms, and its chemical formula was C4511H6895N1187O1370S10. The aliphatic index was 69.36 and the grand average hydropathicity index (GRAVY) was − 0.564, which reflects the vaccine’s polar nature and effective interaction with water, suggesting high solubility. The instability index was calculated at 23.00, which was < 40, classifying the vaccine as a stable protein (Table 5). The solubility of the vaccine construct was 0.460 according to QuerySol (Fig. 3).

**Table 5:** Physicochemical properties of the final vaccine construct

Characteristics	Assessment
Number of amino acids	924
Molecular weight	99 KDa
Theoretical pI	8.13
Total number of positively charged residues (Arg + Lys)	105
Total number of atoms	13,973
Chemical formula	C4511H6895N1187O1370S10
Estimated half-life (mammalian reticulocytes, in vitro), (yeast, in vivo), and (Escherichia coli, in vivo)	1.9, 20 and 10 h
Aliphatic index	69.36
Instability index	23.00
Grand average of hydropathicity (GRAVY)	− 0.564
Solubility	0.460
Antigenicity	0.8952%
Allergenicity	Non-Allergen

### Evaluation of antigenicity, allergenicity and toxicity of protein sequences

The designed multi-epitope vaccine was evaluated for antigenic, non-allergenic and non-toxic properties. The antigenicity of the final vaccine construct was predicted at 0.8952% by VaxiJen at a 0.4% threshold for the bacterial model. Allergenicity and toxicity were evaluated to ensure that the candidate vaccine did not have any allergic reactions or toxic effects after entering the body. As predicted by AllerTOP 2.0 and ToxinPred web servers, the vaccine candidate was non-allergenic and non-toxic.

### Multi-epitope vaccine construction

The multi-epitope vaccine construct was composed of a combination of 37 T cells (18 MHC-I and 19 MHC-II epitopes) and 19 linear B cell epitopes using AAY, GPGPG and KK linkers. All predicted epitopes were carefully selected and shown to be non-allergen, non-toxic, and highly antigenic.

### Population coverage and conservancy of epitopes

**Fig. 4:**
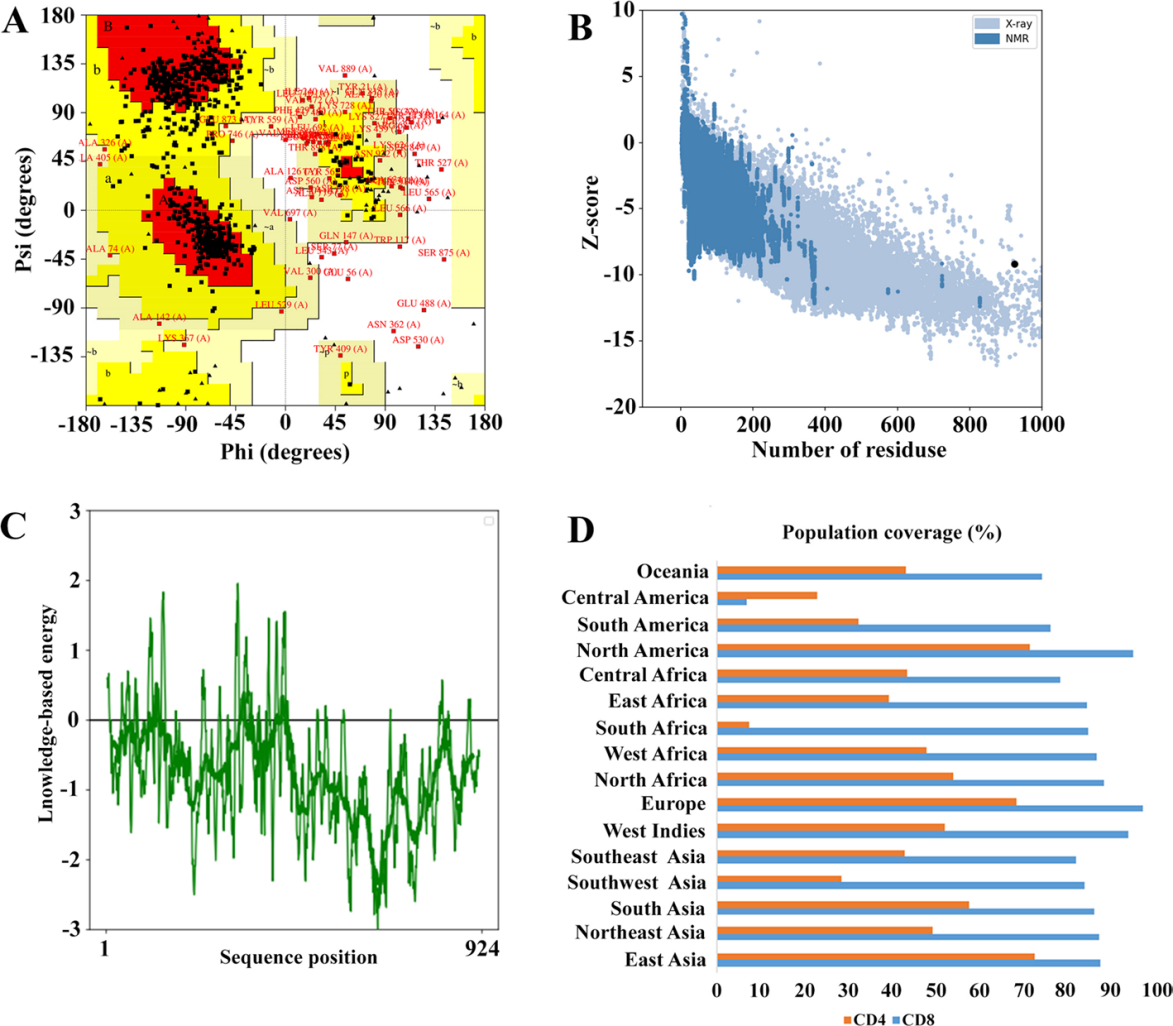
Vaccine 3D Structure Validation by UCLA-DOE LAB and ProSA-web. **A** The statistics of the Ramachandran chart show the most favorable region, additionally allowed, generously and disallowed (outlier) area with 70.7%, 20.8%, 5.8%, and 2.7%, respectively. **B** Based on ProSA-web, the Z-score of the refined model is − 9.2. **C** The server also draws a plot to check the quality of the local model, which negative values indicating that there is no error in the structure of the model. **D** Worldwide population coverage rates based on CD8^+^ T cell epitopes and CD4^+^ T cell epitopes

The potential efficacy of a potential vaccine can be determined by the frequency of distribution of HLA alleles in different ethnicities. In this study, population coverage in CD8^+^ and CD4^+^ T cells was investigated separately as well as their combined effect. The predicted T cell epitopes (CD8^+^ and CD4^+^) were exposed to population coverage in 16 different geographical regions of the world, as shown in Fig. 4D. Analyzes showed that among 18 CD8^+^ T cell epitopes, the highest coverage was in Europe (98.07%), North America (95.61%), and West India (94.69%). After that, North Africa (89.06%), East Asia (88.21%), Northeast Asia (88.03%), West Africa (87.42%), South Asia (86.82%), South Africa (85.42%), East Africa (85.26%), Southwest Asia (84.63%), Southeast Asia (82.77%), Central Africa (78.93%), Oceania (74.80%), South America (76.88%) provided other coverage. While the lowest coverage was related to the region Central America (7.01%).

On the other hand, the population coverage results for CD4^+^ T cell epitopes are shown in Fig. 4D. The highest coverage for CD4^+^ T-cell epitopes was found in East Asia (73.14%), North America (71.89%), and Europe (68.97%). Other results were reported in South Asia (58.10%), North Africa (54.47%), West Indies (52.58%), Northeast Asia (49.82%), West Africa (48.30%), Central Africa (43.89%), Oceania (43.57%), Southeast Asia (43.38%), East Africa (39.63%), and South America (32.67%). The lowest population coverage was for Southwest Asia (28.80%), Central America (23.09%) and South Africa (7.65%).

### Secondary and 3D structure prediction of the vaccine construct

**Fig. 5:**
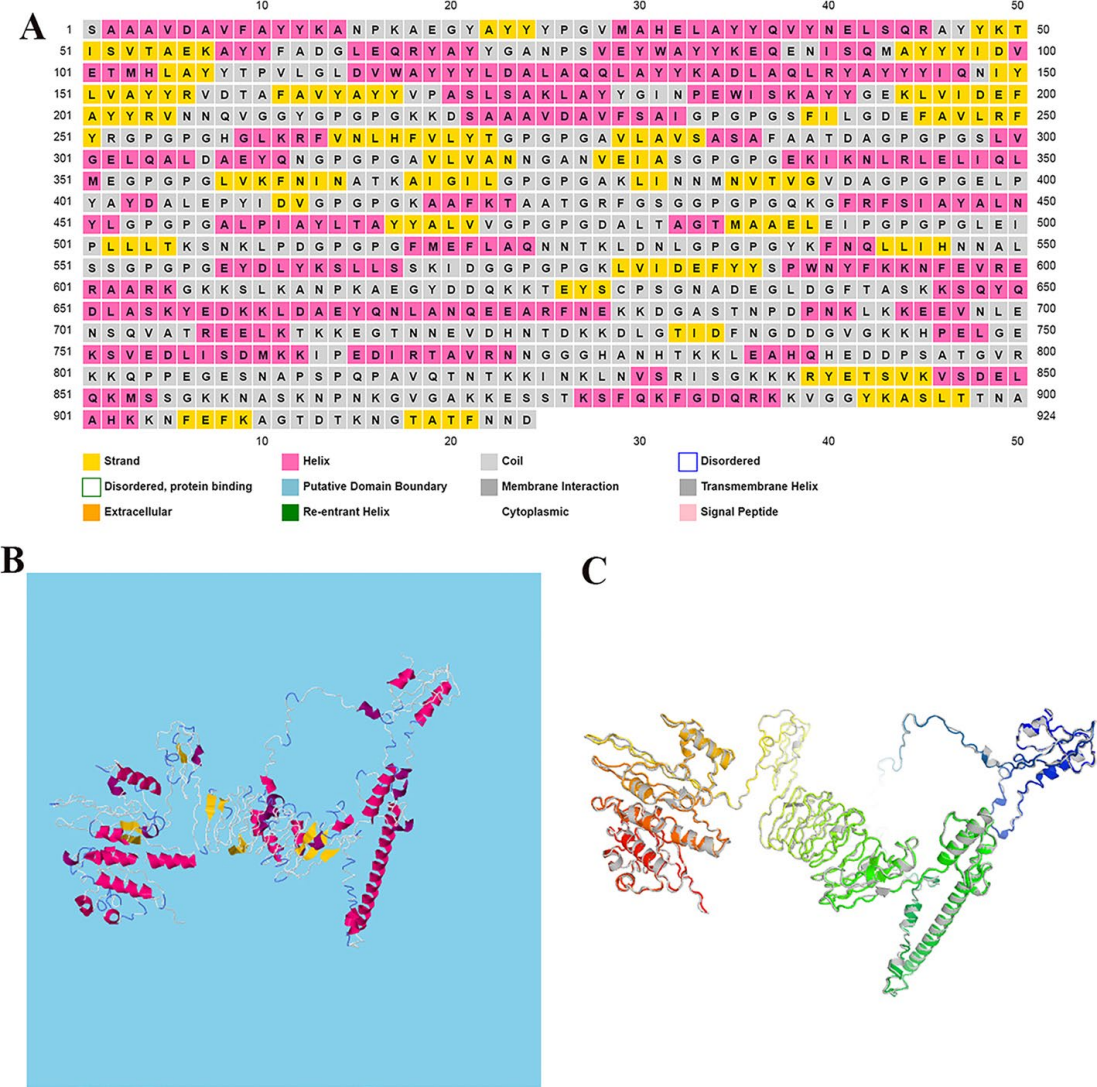
Displays the second and third structures of the final vaccine contracture. **A** In this Figure, the β-strands, the α-helix, and the random coils are shown in yellow, pink, and gray colors, respectively. **B** The 3D structure of a multi-epitope vaccine that was selected as the best model by the RaptorX server. β-strands, the α-helix, and the random coils are shown in yellow, red, and white-blue colors, respectively. **C** The 3D structure of multi-epitope vaccine after refinement

The secondary (i.e. α-helix, β-strand, and random coil) and 3D structure of the final vaccine construct were predicted by PSIPRED and RaptorX servers, respectively. According to the PSIPRED server, the final vaccine contained 34% of the amino acids in the α-helix structure and 16.66% and 49.34% of the amino acids in the β-strand and coil structures, respectively (Fig. 5A).

Five models were suggested by the RaptorX server for 3D structure in PDB format. Among the five models proposed by the RaptorX server, the structure of Model 3 (Fig. 5B) was selected.

### Refinement, validation and quality assessment of the tertiary structure

The GalaxyRefine server was used to increase the overall and partial structural quality of the final vaccine construct. Among the 5 models proposed by this server, the best-refined model (5C) is shown in Fig. 5C with a GDT-HA score of 0.8888, an RMSD score of 0.571, a MolProbity score of 2.614, a Clash score of 32.2 and a Ramachandran score of 88.5 (Table 6). Therefore, it can be concluded that the quality of the refined model is high compared to the raw structure.

**Table 6:** Quality scores of 5 models predicted by GalaxyRefine server

Model	GDT-HA	RMSD	MolProbity	Clash score	Poor rotamers	Rama favored
Initial	1.0000	0.000	5.202	318.7	89.4	77.7
MODEL 1	0.8885	0.576	2.730	32.5	1.5	88.2
MODEL 2	0.8861	0.587	2.586	32.8	1.0	88.5
MODEL 3	0.8883	0.586	2.568	30.3	1.0	87.9
MODEL 4	0.8899	0.588	2.581	31.1	1.0	87.7
MODEL 5	0.8888	0.571	2.614	32.2	1.1	88.5

In validation, Ramachandran diagram analysis based on the PROCHECK server showed that 70.7%, 20.8%, 5.8% and 2.7% of protein residues were located in the most favored region, additional allowed, generously and disallowed (outlier) area of the final vaccine, respectively. (Fig. 4A). The quality and potential errors in the final vaccine 3D model were verified by ProSA-web. The Z-score, which indicates the overall quality of the model, was − 9.2 (Fig. 4B). However, a model with a lower Z-score is considered a higher-quality model. In addition, a plot was drawn to check the quality of the local model, where negative values indicate that there is no error in the model structure (Fig. 4C).

### Protein disulfide bridging for vaccine stability

**Fig. 6:**
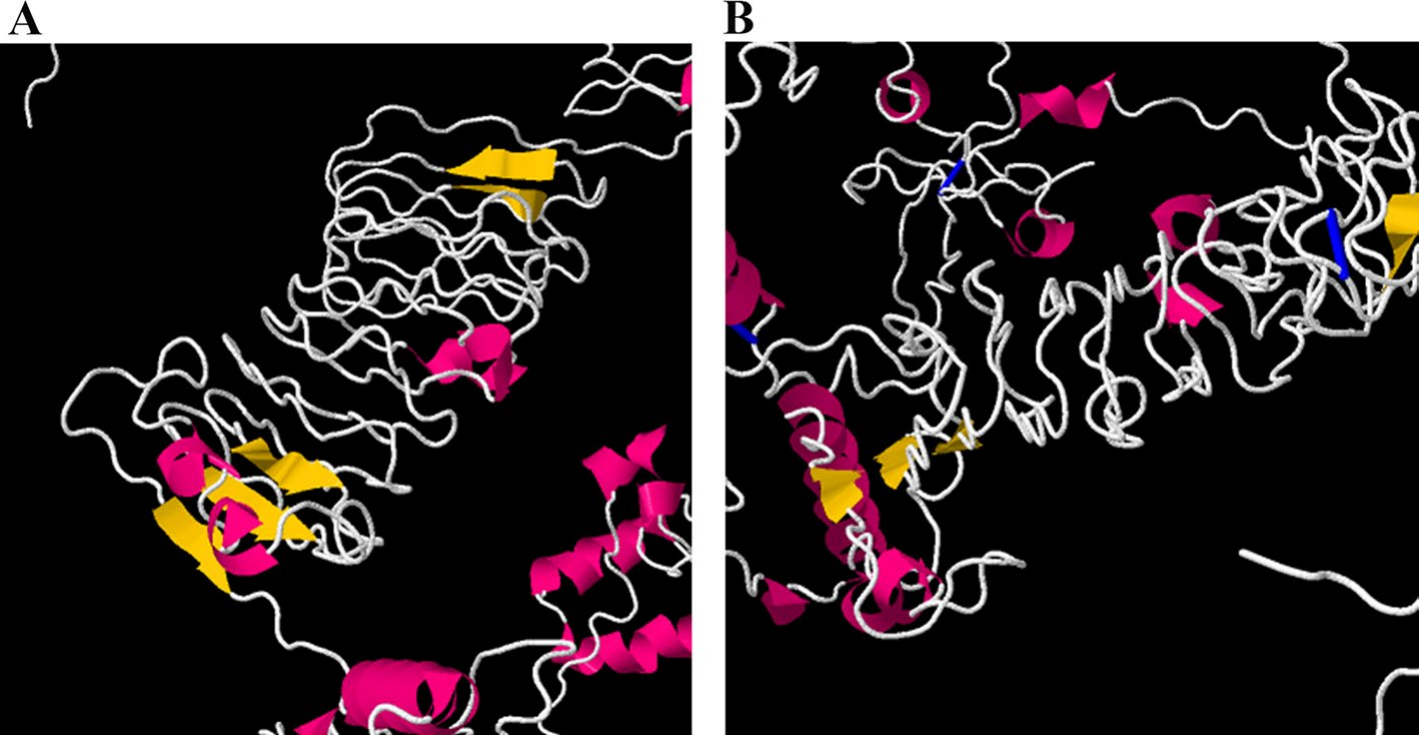
Disulfide engineering display in the final vaccine construct. The original form is shown on the left and the mutant form is on the right

Disulfide engineering was applied to the multi-epitope vaccine construct refined model via DbD2. Four pairs of amino acids from the vaccine construct: 375GLY-377GLY, 642THR-645LYS, 668LEU-702SER, and 826ASN-844VAL, were selected for disulfide bond by the mutation because the bond energy had less than 1 kcal/mol. In addition, these mutations are shown in Fig. 6.

### Molecular docking of multi-epitope vaccine with TLR4 receptor

**Fig. 7:**
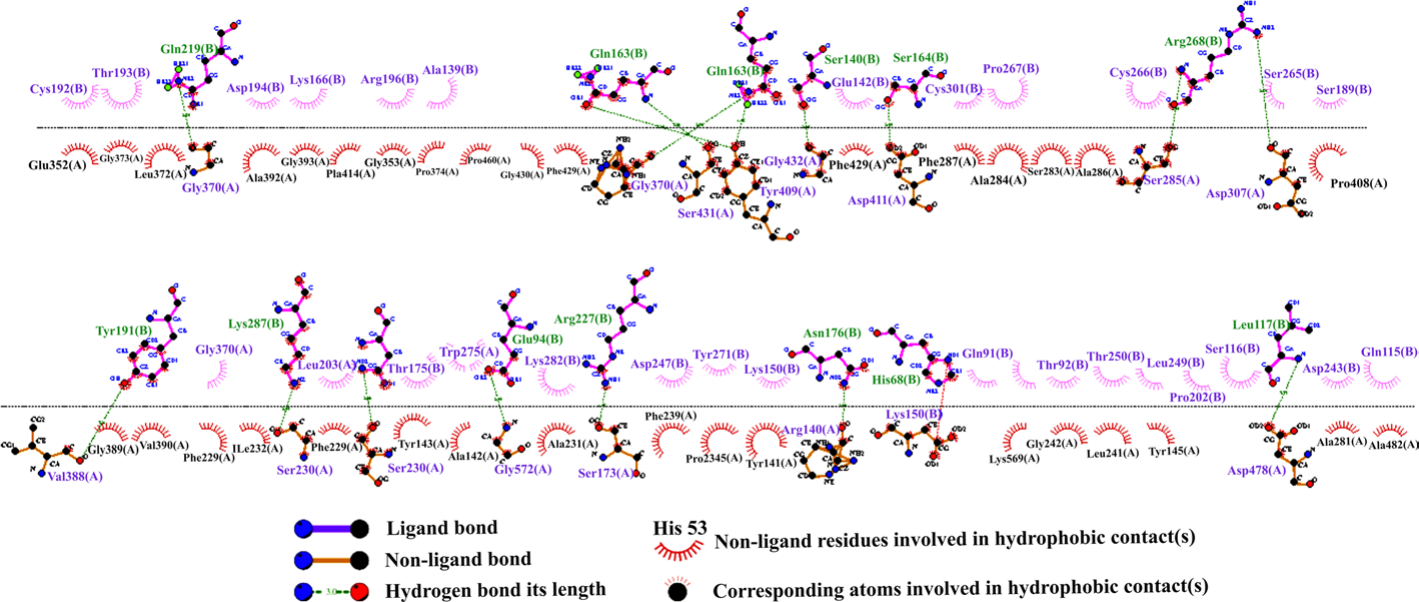
Representation of interacting residues between vaccine docked with TLR4. The Gly370, Arg428, Ser431, Tyr409, Gly432, Asp411, Ser285, Asp307, Val388, Gly233, Ser230, Gly572, Ser173, Arg140, Asp571, Asp478 residues from chain A of the vaccine were bound to Gln219, Gln188, Gln163, Ser140, Arg268, Tyr191, Lys278, Asn279, Glu94, Arg227, Asn176, His68, Leu117 residues from chain B by hydrogen bonds

Molecular docking can evaluate the interactions between a ligand molecule and the receptor molecule to check the stability and binding affinity of their docked complex [84]. In this study, TLR4 was selected as the receptor for molecular binding. The energy scores obtained for best docking the vaccine-TLR4 complex from ClusPro v2.0 and PatchDock servers were − 1232.7 and − 32.40, respectively, which indicates a very good binding affinity. The score of the top models is shown in Table 7. These complexes were subjected to MD simulation to analyze their stability. In addition, a schematic diagram of the interaction between the vaccine structure and TLR4 was created by LigPlot + software (Fig. 7). Hydrogen bonds and salt bridge interactions were obtained by the DIMPLOT program. DIMPLOT was shown, Gly370, Arg428, Ser431, Tyr409, Gly432, Asp411, Ser285, Asp307, Val388, Gly233, Ser230, Gly572, Ser173, Arg140, Asp571, Asp478 residues from chain A of the vaccine were bound to Gln219, Gln188, Gln163, Ser140, Arg268, Tyr191, Lys278, Asn279, Glu94, Arg227, Asn176, His68, Leu117 residues from chain B by hydrogen bonds with bond lengths of 2.84 angstroms (Ǻ), 2.96 Ǻ, 3.26 Ǻ, 1.48 Ǻ, 2.94 Ǻ, 2.55 Ǻ, 2.54 Ǻ, 2.85 Ǻ, 3.21 Ǻ, 2.44 Ǻ, 2.66 Ǻ, 2.55 Ǻ, 1.5 Ǻ, 2.66 Ǻ, 3.33 Ǻ respectively. Also, the Asp571 residue from chain A of the vaccine binds to His68 residue from chain B of the TLR4 by salt bridge interaction.

**Table 7:** Top models of docked complexes of designed vaccine with TLR4

Cluster	Members	Representative	Weighted score
0	147	Center	− 843.1
		Lowest energy	− 1232.7
1	68	Center	− 875.7
		Lowest energy	− 1098.3
2	65	Center	− 950.8
		Lowest energy	− 1080.8
3	60	Center	− 1241.8
		Lowest energy	− 1251.3
4	56	Center	− 934.1
		Lowest energy	− 978.6
5	49	Center	− 863.9
		Lowest energy	− 1174.1
6	26	Center	− 856.9
		Lowest energy	− 926.3
7	22	Center	− 844.5
		Lowest energy	− 949.0
8	18	Center	− 993.8
		Lowest energy	− 993.8
9	17	Center	− 935.4
		Lowest energy	− 935.4
10	16	Center	− 856.4
		Lowest energy	− 946.8

### Molecular dynamic simulation

**Fig. 8:**
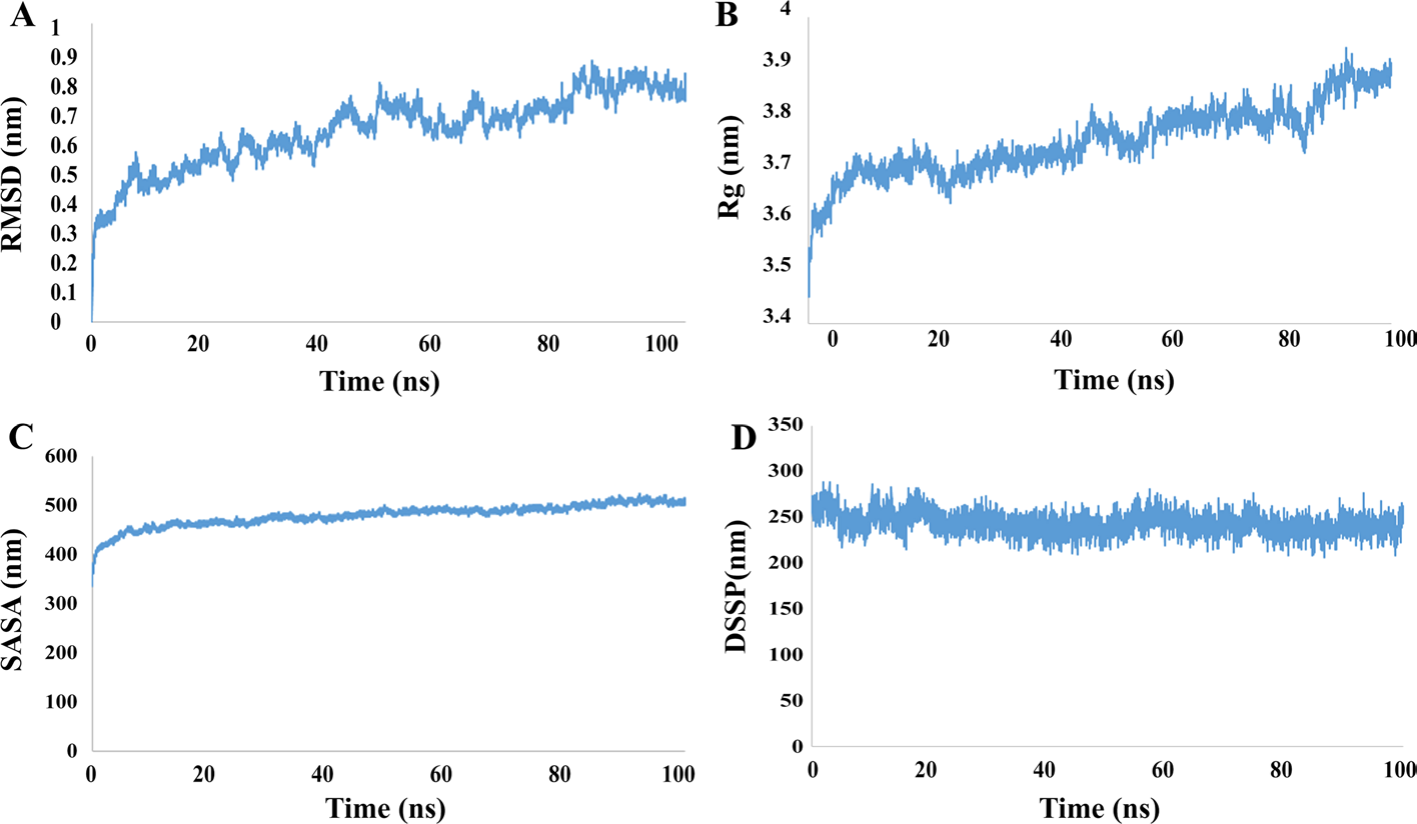
The final construct of the molecular dynamics simulation vaccine with GROMACS software

To evaluate the dynamic properties of the final vaccine, MD simulation was performed for 100 ns the results are represented in Fig. 8. At first, it is important to ensure that the simulation time is sufficient for a particular system. The best method for this is to measure the RMSD of the system during MD simulation. The results of this analysis is showed in Fig. 8A and as it is clear the protein reached its equilibrated state at the time of 50 ns was which followed by some fluctuations in diagram and this confirms that the simulation time is enough for this system. Also it can be seen that there is no sever fluctuation in RMSD diagram which is an index for structural stability of the as designed vaccine. Another issue that might be considered in order to vaccine retain its function is ensuring that the protein is not compressed and the epitopes are not inaccessible. Analyzing the radius of gyration (Rg) of protein is used in MD to evaluate time dependence changes in compactness of its structure. Figure 8b shows the changes in Rg for the designed vaccine during the simulation. As can be seen, the value of Rg is increased for the protein which indicated that its conformation is expanded after simulation. Another confirmation for this can be achieved by analyzing the value of solvent accessible surface area (SASA). The result of SASA analysis is reported in Fig. 8c and as it is clear in its diagram, the surface area is increased along the simulation time. Together with Rg analysis, these results suggest that the structure of protein did not undergo compactness and this prevent disabling the vaccine epitopes. Another important factor for vaccine Immuno-modulation is stability in its secondary structures which can be investigated by an analysis called DSSP. Figure 8d shows the changes in protein secondary structures during the simulation time. After 100 ns of MD simulation there is just less than 8 percent of protein residues which undergoes denaturation from their secondary structures. This predicts structural stability of the designed vaccine under the similar condition to which it may be assigned. In conclusion the results of MD simulations confirm that the as designed vaccine maintain its functional state in solution and can be tested for its Immuno-modulation ability in experimental.

### NMA evaluation of the vaccine-receptor complex

**Fig. 9:**
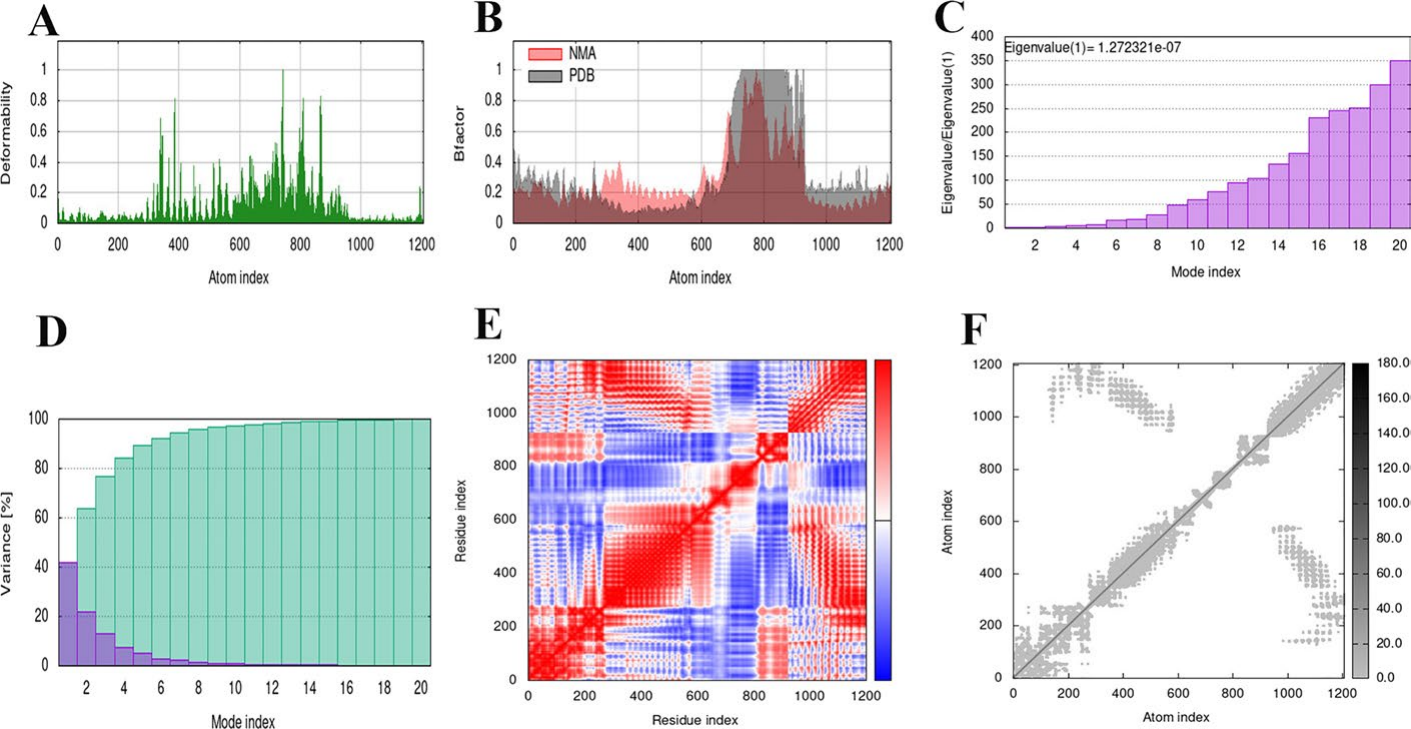
The Molecular dynamics simulation of the vaccine–TLR4 complex. Six graphs including **A** Deformability index, **B** B-factor values calculated by normal mode analysis, **C** The eigenvalue of the docked complex, **D** The covariance matrix between pairs of residues, **E** The elastic network model are shown, **F** The darker the gray dots, the greater the protein stiffness in certain sections

NMA was conducted to scrutinize protein stabilization and their large-scale mobility [92]. MD simulation of the vaccine candidate/TLR4 interactions is shown in Fig. 9. Figure 9A shows the deformation of the protein flexibility, which depends on the individual distortion of each residue depicted by the chain hinges. On the other hand, locations with hinges are areas with high deformability and illustrate a stable binding. The b-factor shows the relative amplitude of atomic displacement for the equilibrium position. According to Fig. 9B, few fluctuations of atomic displacement were observed for the TLR4-vaccine complex. Figure 9C showed the eigenvalue determined for the complex, which was 1.272 e-07. This Figure also showed that it has relatively least energy required to deform its structure based on the lowest eigenvalue. Figure 9D shows the variance plot of the complexes. In this diagram, the variance associated with the eigenvalue is inversely related to the individual variance shown by the blue-colored bands and the cumulative variance shown by the green bands (Fig. 9D). Figure 9E shows a covariance matrix map of the interaction between residue pairs of the proteins of a complex (red: correlated motion between a pair of residues, white: non-correlated motion, and blue: anti-correlated motion). Finally, the stiffness study of the protein complex was performed using elastic network analysis. As shown in Fig. 9F, the darker the gray dots, the greater the protein stiffness in certain sections.

### Immune simulation

**Fig. 10:**
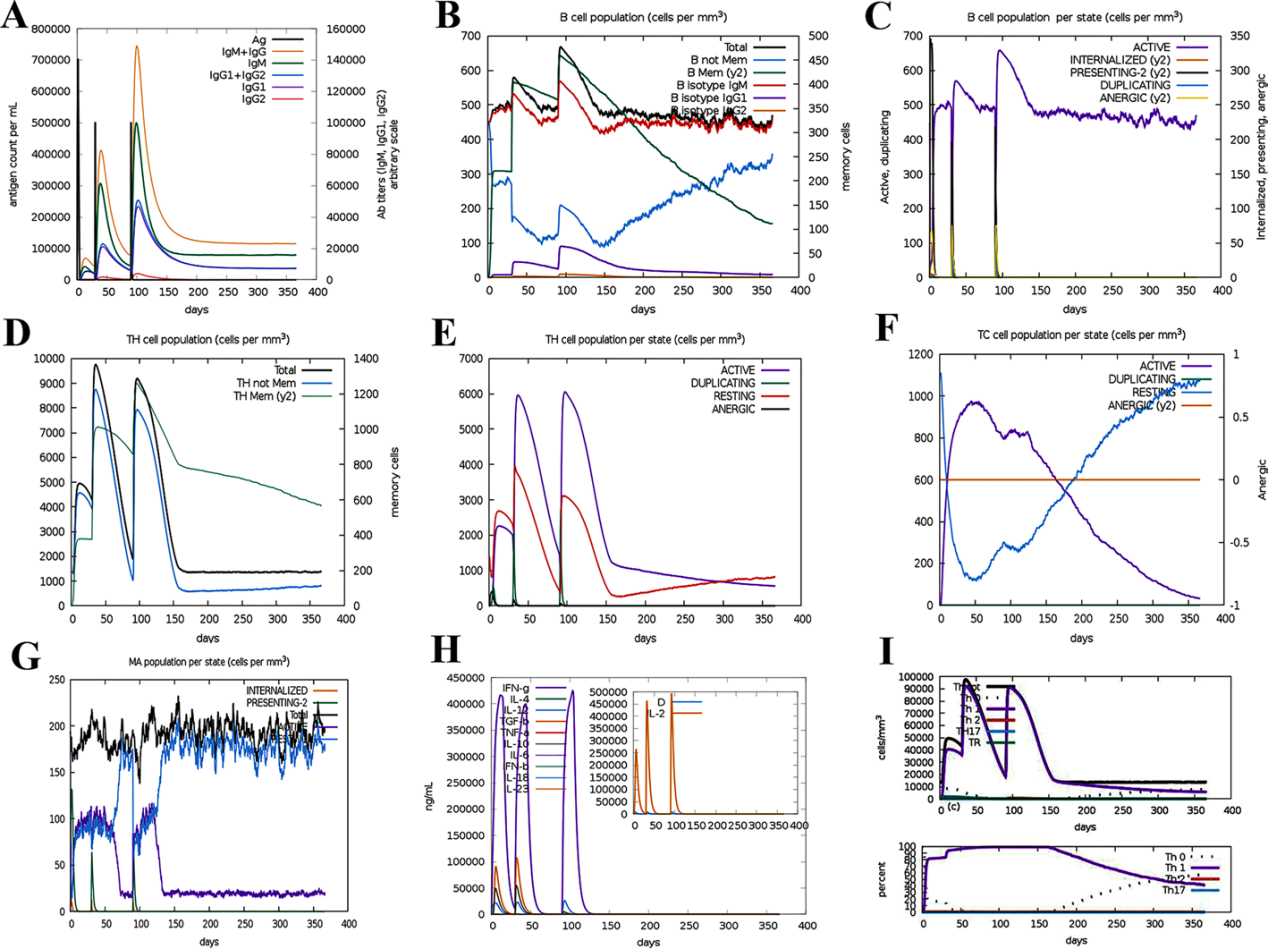
Immunization simulation results by C-ImmSim of the construct of multi-epitope vaccine as an antigen. **A** Demonstration of immunoglobulin production in response to antigen injection after vaccine administration, shown as different color peaks. **B** B cell population after three vaccine injections, which indicates an increase in different types of B cells and their class-switching potential. **C** Displays the population results per state of B cell. **D** The evolution of T-helper cells. **E** Population per state of T-helper cell. **F** Production of cytotoxic-T cells after vaccine injection. **G** Macrophages population per state. **H** Induction of cytokines and interleukins (increased production of IFN-γ and IL-2) after vaccination. **I** Th1-mediated immune response

The multi-epitope vaccine designed to evaluate the specific immune response of the vaccine was submitted to the C-IMMSIM v10.1 server. Secondary and tertiary immune responses showed higher levels of antibodies (IgM + IgG, IgG1 + IgG2, IgM, IgG1) than the primary immune response, which coincided with a decrease in antigen levels (Fig. 10A). In addition, several long-lasting B cell isotypes were found, indicating possible isotype switching potentials and memory B cell formation (Fig. 10B). On the other hand, Fig. 10C shown the increase in cell proliferation in B cells as well as the presentation of antigens after vaccination. According to Fig. 10D–F, the levels of TH (helper) and TC (cytotoxic) cell populations also increased significantly in memory development. Increased macrophage activity and antigen presentation are shown in Fig. 10G. Figure 10H shows a significant increase in interferon-gamma titer as well as a moderate increase in interleukin 2 (IL-2) (Fig. 10H) after the third injection of the vaccine. Finally, we saw a significant increase in Th1 (Fig. 10I). All of these data suggest that our candidate multi-epitope vaccine can induce an effective immune response that can protect against pathogens.

### *Codon adaptation and *in silico* cloning*

**Fig. 11:**
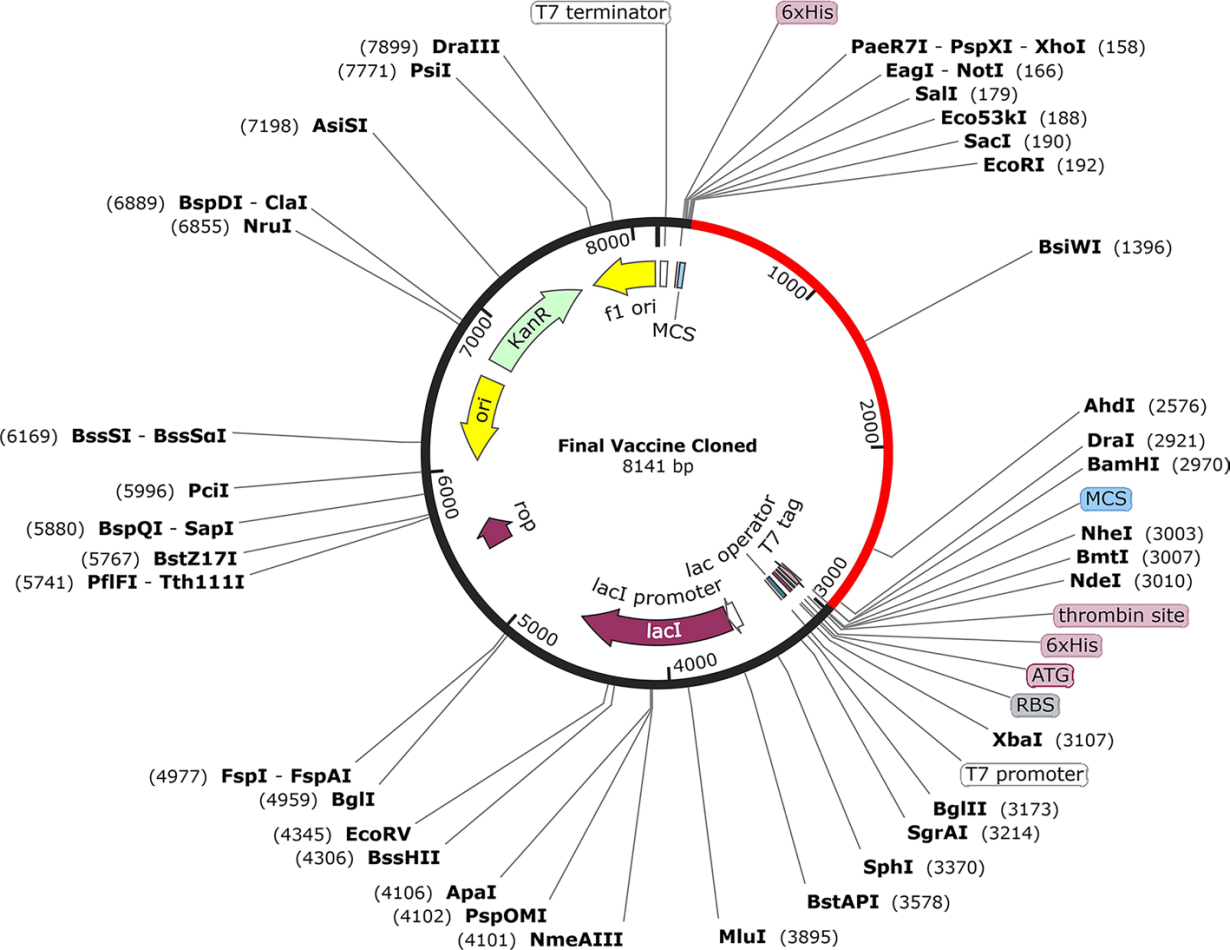
In silico cloning of the final vaccine construct into pET28a (+) expression vector. The vector was shown in black color, while the red color provided the gene coding for the vaccine to construct a protein. *EcoRI* and *BamHI* restriction enzyme sites have been proposed as cutting sites

The CAI and GC content of the long nucleotide sequence of 2772 bp was evaluated to optimize the vaccine construct. Better expression (transcription and translation) in organisms requires a GC content between 30 and 70% to be optimal, while a CAI value should be higher than 0.8 to 1 [93, 94]. The GC content and CAI values of the optimized nucleotide sequence obtained from the Jcat server were 50.180% and 0.9913, respectively. *EcoRI* (GAATTC) and *BamHI* (GGATCC) restriction sites were added to the N and C terminals of the final vaccine codon sequence. Finally, SnapGene software was used to integrate the adapted DNA sequence to the pET-28a (+) vector, between the *EcoRI* and *BamHI* restriction sites (Fig. 11).

### MRNA prediction of the designed vaccine

The secondary structure of the vaccine MRNA sequence was predicted by the RNAfold server with a minimum free energy score of − 861.60 kcal/mol. A lower MFE indicates a higher thermodynamic stability of the MRNA secondary structure.

## Discussion

CRC is known as the fourth cause of death among cancers and predisposing factors like different lifestyle, genetic and environmental risk reasons can promote the cancer [95]. CRC is induced by normal epithelium alteration into high-proliferative epithelial cells and results in the reorganization of intestinal epithelial cells and adenoma-carcinoma formation. This cancerous process metastasized to the colon and may progress to CRC [96]. There is a strong association between the presence of *S. bovis, B. fragilis, H. pylori, F. nucleatum, E. faecalis, E. coli, and P. anaerobius* and the incidence of CRC [1, 14, 95]. Even with antibiotic treatment, there is a high risk for the recurrence of disease and the emergence of antibiotic-resistant strains, so there is a need to develop novel methods like immunization via vaccines against pathogenic and toxigenic strains [97].

Peptide-based vaccines, especially those contain cocktail of several peptides, show a significant effect on the treatment outcome of patients with CRC [98]. These vaccines target either host proteins [99] or immunogen proteins of pathogens related to cancer [100].

A desirable multi-epitope vaccine should be consisting of peptides with capable of generating CTL, TH and B cells and triggering potential immune response against [101]. Today, the design of multi-epitope vaccines is recognized as an emerging area that is of considerable importance. However, vaccines designed with this approach have been shown in vivo efficacy with protective immunity, but have also entered phase I clinical trials [102–105].

This study aimed to design a multi-epitope and prophylactic vaccine against colorectal cancer-related pathogens based on the immunoinformatics approach. Considering the importance of the virulence factors, 10 proteins from different microorganisms were selected to predict effective epitopes.

A total of 56 epitopes (924 amino acids) including 19 epitopes for B cell, 19 epitopes for MHC I binding, and 18 epitopes for MHC II binding were considered. For the epitope to be effective and safe for the host, it must be antigenic, non-allergenic, non-toxic, and stable. All the selected epitopes were antigenic, non-allergenic and non-toxic.

The suitable molecular weight of the designed vaccine makes it easy for purification, so can be considered a suitable vaccine. Higher aliphatic index values indicate greater thermostability at several temperatures and negative GRAVY values indicate the hydrophilic nature of the candidate vaccine, so it can show strong interactions with water molecules. The vaccine instability index was calculated to be 23.00 and since it was less than 40.00, it was considered a stable protein. Also, the designed vaccine has good solubility.

PSIPRED and RaptorX web servers were used to evaluate the structure of the second and 3D candidate multi-epitope vaccines, respectively. Accordingly, the PSIPRED server predicted the α-helix, β-strand, and coil of the candidate vaccines to be 34%, 16.66%, and 49.34%, respectively.

In the final constriction refining, five proposed refined models were introduced and model 5 was selected as the best-refined model with GDT-HA 0.8722, an RMSD score of 0.586 a MolProbity score of 2.582, a Clash score of 32.1, and a Ramachandran score of 88.3. Studies have shown that RMSD < 2.0 Å corresponds to good docking solutions [106], and in the present study, our final RMSD construct was also in the best condition.

Because disulfide bonds play an important role in folding, stability, and protein function, if they are ignored, the stability of the target protein can be reduced [107]. For this reason, we saw 4 pairs of 375GLY-377GLY, 642THR-645LYS, 668LEU-702SER, and 826ASN-844VAL amino acids with less than one energy bond, which indicates more stability of the final construct.

Different frequencies of HLA type vary in different ethnicities around the world due to the high polymorphism of the MHC molecule. The selected alleles considered in this study proved to show sufficient population coverage a large scale (Fig. 4D). The highest population coverage in CD8^+^ T cells is in Europe (98.07%), North America (95.61%), and West India (94.69%). While the lowest CD8^+^ T cells population coverage is in Central America (7.01%). On the other hand, the largest population coverage in CD4^+^ T cells is in East Asia (73.14%), North America (71.89%), and Europe (68.97%). The lowest population coverage was for Southwest Asia (28.80%), Central America (23.09%) and South Africa (7.65%).

Two online servers, ClusPro 2.0 and PatchDock & FireDock were used for docking analysis to increase our forecast accuracy. These servers pointed to a strong interaction between the TLR4 and the designed vaccine. The energy scores obtained for binding the vaccine-TLR4 complex using these two servers indicated a very good binding affinity. In summary, the MD simulation findings obtained from the present study confirm that the designed vaccine molecule can interact optimally with the TLR4 protein. The C-IMMSIM server was then used to evaluate the ability of the candidate vaccine to initiate an immune response with an immune simulation. However, based on the results, enhancement of memory B cells and T cells was visible. Also, the secondary and tertiary immune responses showed higher levels of antibodies than the primary immune response. On the other hand, a significant increase in IFN-γ titer as well as a moderate increase in IL-2 was shown after the third injection of the vaccine. All these data suggest that our candidate multi-epitope vaccine can induce an effective immune response that can protect against pathogens.

Finally, to ensure the translation efficiency of the multi-epitope vaccine designed in a specific expression system, the vaccine MRNA was amplified using the JCAT. Adaptive DNA sequences between *EcoRI* (GAATTC) and *BamHI* (GGATCC) restriction enzyme cleavage sites were then added to N and C terminals, respectively, and subsequently cloned into pET28a (+), the expression vector. The codon adaptability index (0.98) and GC content (53.63%) were promising for the expression of high-level proteins in bacteria. On the other hand, during predicting the stability of the secondary structure of the vaccine mRNA, the RNAfold server produced less negative and less free energy, so it can be concluded that the predicted vaccine can be stable after in vivo transcription.

## Conclusion

CRC is one of the most common cancers worldwide. Increasing evidence suggests that gut microbiota dysbiosis is closely related to CRC. *Streptococcus bovis*, *Helicobacter pylori*, *Bacteroides Fragilis*, *Fusobacterium nucleatum*, *Enterococcus faecalis*, *Escherichia coli*, and *Peptostreptococcus anaerobius* are the main microbial agents involved in CRC pathogenesis. Therefore, in the present study, an in silico vaccine was designed against their most important epitopes, then its effectiveness was evaluated through different immunoinformatics servers. The designed multi-epitope vaccine seems to act as an effective prophylactic candidate vaccine since the results showed an increase in antibodies, T lymphocytes, and its subtypes (such as helper T lymphocytes and cytotoxic T lymphocytes) as well as INF-γ levels. In general, the application of these results is pending validation in the wet lab experimental models.

## Limitations

Each of these bioinformatics predictive servers has limitations that are not comparable to the experimental method. For example, the C-IMMSIM server simulator is limited because it does not have the disease layer and is unable to detect vaccine efficacy. On the other hand, NMA is probably the least computationally expensive method for studying the dynamics of macromolecules, but the MD method is more accurate than NMA.

A major limitation of this study is the lack of the experimental validation and evaluation of the safety and efficacy of the designed vaccine construct. However, major steps such as laboratory and animal studies are needed to justify our findings to determine safety, efficacy, and immunogenicity as a possible preventive measure. In general, the application of these results is pending validation in the wet lab experimental models.

## Data Availability

All the data supporting the findings are contained within the manuscript.
